# Severe aortic regurgitation in a quadricuspid aortic valve: A false rheumatic disease

**DOI:** 10.1002/ccr3.2060

**Published:** 2019-02-19

**Authors:** Carima Belleyo‐Belkasem, Alejandro de La Rosa‐Hernández, María Manuela Izquierdo‐Gómez, Javier García‐Niebla, Juan Lacalzada‐Almeida

**Affiliations:** ^1^ Department of Cardiology Hospital Universitario de Canarias Tenerife Spain; ^2^ Servicios Sanitarios del Área de Salud de El Hierro Valle del Golfo Health Center El Hierro Spain

**Keywords:** quadricuspid aortic valve, transesophageal echocardiography

## Abstract

The quadricuspid aortic valve (QAV) is a malformation that leads to severe valve failure later in life. Malformation and displacement of coronary ostia are found in 10% of patients. In this context, transthoracic echocardiography (TTE) and transesophageal echocardiography (TEE) are the essential imaging tests for the preoperative assessment for cardiac surgery.

## 1 QUESTIONS AND TEXT

What is the natural history of the quadricuspid aortic valve (QAV)?

A. The QAV rarely leads to aortic regurgitation.

B. This valvulopathy in most cases does not progress with time.

C. The QAV leads to regurgitation in most cases, even when the 4 cusps are equal.

D. The QAV must be repaired or replaced in most cases because of severe stenosis.

The correct answer is C.

**Figure 1:**
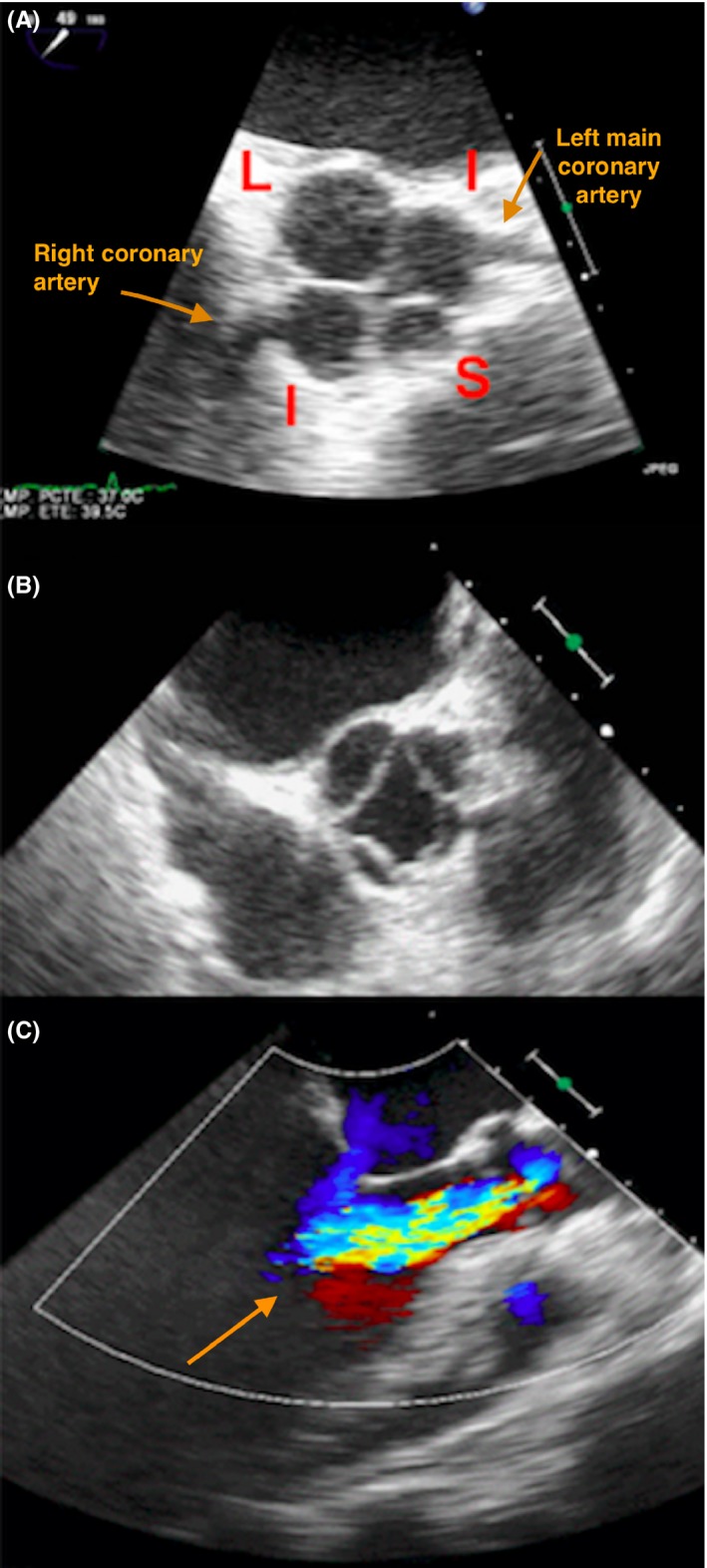
Views of the transesophageal echocardiogram: A, Mid‐esophageal short‐axis view shows the aortic valve in diastole with 4 cusps: one large (L), one small (S), and two intermediate (I) cusps. The small cusp (S) is the non‐coronary cusp. The large cusp (L) is the supernumerary cusp. B, Mid‐esophageal short‐axis view shows the quadricuspid aortic valve in systole. C, Mid‐esophageal long‐axis color flow view showing a severe aortic insufficiency (see arrow)

**Figure 2:**
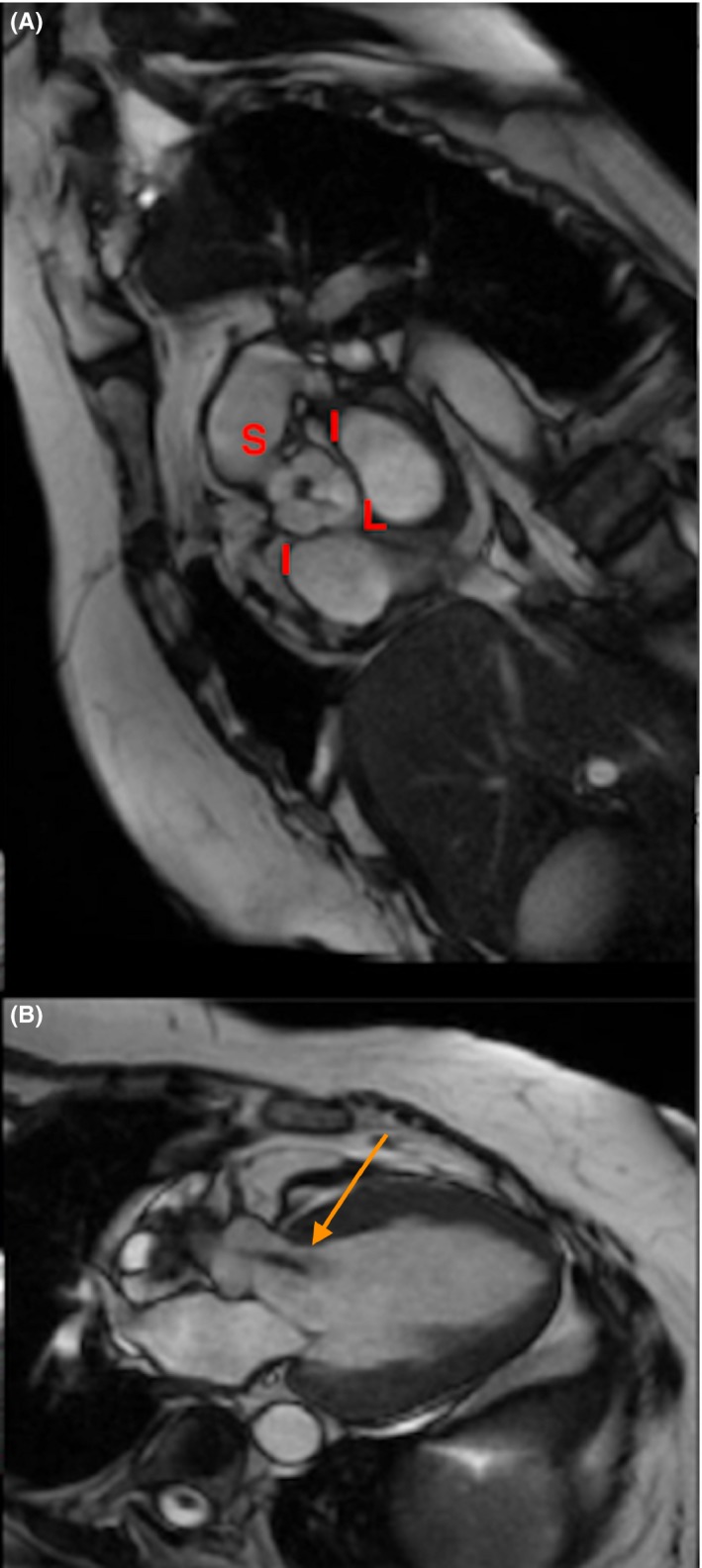
Cardiac magnetic resonance images. A, Short‐axis cine of aortic valve shows a central non‐coaptation of a quadricuspid aortic valve type D (four cusps: one large (L), one small (S), and two intermediate (I)) according to the Hurwitz & Roberts classification. B, The three chamber view in end‐diastole shows a dilated left ventricle and non‐quantifiable aortic valve regurgitation (see arrow)

**Figure 3:**
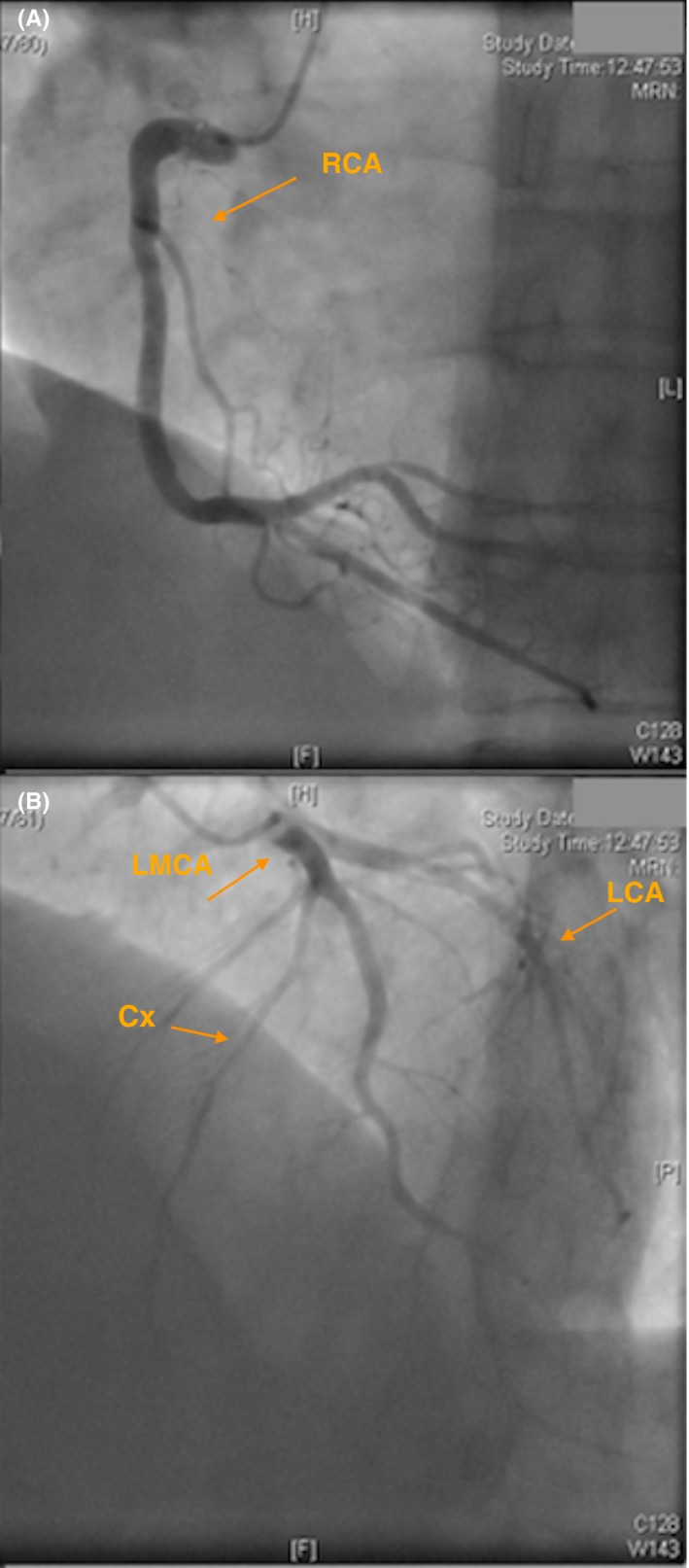
Coronary angiography. A, Right anterior oblique caudal projection displays the right coronary artery (RCA). B, Projection with left anterior oblique cranial angulation displaying the left main coronary artery (LMCA) with an early division in the left anterior descending (LAD) artery and the circumflex (Cx) coronary artery

A 62‐year‐old Caucasian female presented with acute heart failure. She was previously asymptomatic with chronic moderate aortic regurgitation, a valvulopathy that had previously been labeled as rheumatic. The TTE showed a dilated left ventricle with severe dysfunction and severe aortic regurgitation. TTE imaging was suboptimal in analyzing the valve morphology. TEE is a useful tool for the morphological diagnosis of AV disease and for ruling out congenital anomalies of the coronary arteries, which are the most frequent congenital anomalies associated with QAV.^1^ TEE images revealed a QAV type D with severe regurgitation according to the Hurwitz and Roberts anatomical classification system.^2^ The aortic valve did not show the thickening that is typical of rheumatoid valve disease or commissural fusion of the leaflets. There were four cusps: one large (L), two intermediate (I), and one small (S) cusp (Figure 1A,B, and C). Cardiac magnetic resonance imaging showed the same findings as the TEE; namely, a QAV with severe aortic regurgitation (Figure 2A,B). The angiogram excluded coronary artery anomalies before the AV replacement (Figure 3A,B).

## CONFLICT OF INTEREST

None declared.

## AUTHOR CONTRIBUTION

JLA: involved in conception and design of the work. JGN, CBB, and ARH: wrote the case description and involved in critical revision of the work. MMIG: contributed to the description of echocardiography and cardiac magnetic resonance. ARH: provided the description of the coronary angiography and the key clinical message. All authors were involved at each stage of the revision process and contributed substantially to the project's intellectual content.
